# Lung Function Parameters during Anti-TNFα Therapy in Inflammatory Bowel Disease

**DOI:** 10.2174/0118743064437075260515051440

**Published:** 2026-05-25

**Authors:** Maximilian Schulze, Bernadette Schulze, Gero Moog, Serap Alp Bastian, Andreas Bastian

**Affiliations:** 1 Department of Internal Medicine, Kantonsspital St.Gallen, St.Gallen, Switzerland; 2 Medical Center Rappjmed AG, University of Applied Sciences Rapperswil, Rapperswil-Jona, Switzerland; 3 Department of Gastroenterology, Marienkrankenhaus Kassel, Kassel, Germany; 4 Department of Pneumonology, Intensive Care Medicine and Infectiology, Marienkrankenhaus Kassel, Kassel, Germany

**Keywords:** Lung function parameters, FEV1, VC, RV, DLCO SB, Anti-TNFα therapy, Inflammatory bowel disease (IBD)

## Abstract

**Introduction:**

Patients with Inflammatory Bowel Disease (IBD) may experience a range of extraintestinal pulmonary manifestations. Anti-TNFα therapy has emerged as a crucial treatment option for IBD patients. Despite the pro-inflammatory role of TNFα in pulmonary diseases, anti-TNFα therapy has no major value in their treatment. Therefore, this study aimed to investigate the prevalence of pulmonary manifestations and the impact of anti-TNFα therapy on lung function parameters in IBD patients.

**Methods:**

Thirty two patients with IBD were recruited. These patients received pulmonary function tests prior to and at least 6 weeks after initiating anti-TNFα therapy. Pulmonary function was evaluated by standardized body plethysmography, including the measurement of lung diffusion capacity (DLCO SB).

**Results:**

FEV1 and vital capacity (VC) were within normal range before and during anti-TNFα therapy. Mean residual volume (RV) was moderately increased before initiation of anti-TNFα therapy. RV decreased during the course of therapy, from 2.7 L (interquartile range (IQR) 2.04 - 3.06) or 159% (IQR 129.25 - 184.75) prior to anti-TNFα therapy to 2.5 L (IQR 2.14 - 3.00) or 138% (IQR 127.00 - 165.25) during anti-TNFα therapy (*p*=0.031 for absolute values and *p*=0.014 for relative values. DLCO SB was slightly reduced before anti-TNFα therapy and decreased even further during anti-TNFα therapy, from 76.1% to 72%.

**Discussion:**

Lung function tests in our IBD cohort were almost normal. FEV1 and VC were within normal range. RV was moderately increased, and DLCO SB was slightly decreased. These might indicate an extraintestinal involvement of IBD. During anti-TNFα therapy, RV slightly improved, whereas DLCO SB decreased even further, albeit only slightly. In our IBD cohort, anti-TNFα therapy did not significantly influence lung function parameters.

**Conclusion:**

Our study shows that anti-TNFα therapy in Inflammatory Bowel Disease (IBD) only slightly changes lung function parameters, including diffusion capacity, at least in patients with mild IBD.

## 1 INTRODUCTION

Inflammatory bowel diseases, including ulcerative colitis and Crohn's disease, have developed into global diseases, with increasing incidence in many parts of the world and the highest prevalence in Europe [1]. The exact aetiology is still unknown, but it is assumed to be a multifactorial disease with a complex interplay among environment, genetics, microbiology, and immunology [2, 3]. Extraintestinal manifestations are observed in up to 50% of all IBD patients, with the potential to affect almost any part of the body [4]. Consequently, the presence of lung manifestations in IBD patients is described as well [5]. Pulmonary manifestations are very diverse and comprise large airway diseases, small airway diseases, and parenchymal disorders [6–8]. In body plethysmography examinations of IBD patients, significant reductions in FEV1, FVC, FEF 25%/50%/75%, RV, RV/TLC, and the diffusing capacity are observed compared with the reference range [9, 10]. Especially, DLCO is often significantly decreased and is also described as the only changed parameter [11, 12]. Despite an improvement in DLCO value during the remission phase of IBD patients, the value remains reduced compared to the control group [12, 13]. This may indicate a correlation between disease activity and alveolar inflammation, as an extraintestinal manifestation [12]. The observation of elevated lymphocyte levels in the sputum of IBD patients could support this assumption. Like DLCO, the lymphocyte count is elevated to a greater extent during the active phase of the disease than during remission [14].

Anti-TNFα therapy has become a key treatment for IBD. European guidelines recommend the use of this treatment in many cases of moderate to severe disease activity, or as a second-line treatment after unsuccessful previous therapy [15–17]. Besides the intestines, TNFα has also been identified as a pro-inflammatory factor in the lungs. Elevated levels of TNFα have been observed in patients with idiopathic pulmonary fibrosis [18], as well as in the sputum of patients with COPD and asthma [19–21]. Furthermore, there is a probable involvement in lung diseases such as pulmonary emphysema and pneumonia [22]. The use of anti-TNFα therapy in sarcoidosis has been shown to improve pulmonary function tests [23]. However, anti-TNFα therapy may also have a detrimental impact on the lungs. A higher mortality rate has been demonstrated in patients with rheumatoid arthritis and interstitial lung disease (RA-ILD treated with anti-TNFα therapy) [24]. Moreover, the risk of tuberculosis is elevated in patients undergoing treatment with TNFα antagonists [25, 26]. Furthermore, cases of infliximab-induced interstitial pneumonia have been reported [27]. Currently, anti-TNFα agents are considered a secondary treatment option for lung diseases.

## 2 METHODS

### 2.1 Trial Design and Patient Characteristics

A single-center observational trial was conducted to assess the effects of anti-TNFα therapy on lung function in patients with IBD. The data collection covered the period from August 2017 to December 2020 at Marienkran-kenhaus Kassel. The institutional ethics committee of Marienkrankenhaus Kassel, Germany, approved the study (reference no. MKH 05/2017). All patients gave written informed consent for the scientific use of their clinical data.

A total of 32 patients were enrolled prior to the commencement of anti-TNFα therapy for the treatment of inflammatory bowel disease. Two body plethysmographies were performed, including a measurement of the diffusion capacity. The initial body plethysmography was conducted immediately before the commencement of anti-TNFα therapy with infliximab. The second examination was conducted after at least 6 weeks of anti-TNFα therapy. During this time, patients received at least 2 doses of infliximab. All patients had been diagnosed with inflammatory bowel disease.

Patients included in this study were between 18 and 53 years of age. Their mean age was 33 years; most patients were male (male=20, female=12), and more patients with MC participated (MC=18, CU=14) (Table **1**). Exclusion criteria included age < 18 years, pregnancy, and any known pulmonary diseases other than asthma.

### 2.2 Lung Function Testing

Patients underwent standardized body plethysmo-graphy, including measurement of diffusing capacity (MasterScreen™ Body, JAEGER).

For our study, the lung function parameters, vital capacity (VC), forced expiratory volume (FEV 1), residual volume, diffusing capacity (DLCO), and alveolar volume (Va), were recorded. A comparison was made between the lung function tests performed before and during the anti-TNFα therapy. Furthermore, the mean values of the lung function tests were compared with the age, weight, height, and sex-adjusted reference values, derived from a cross-sectional study of the healthy population.

## 3 RESULTS

**Fig. (1):**
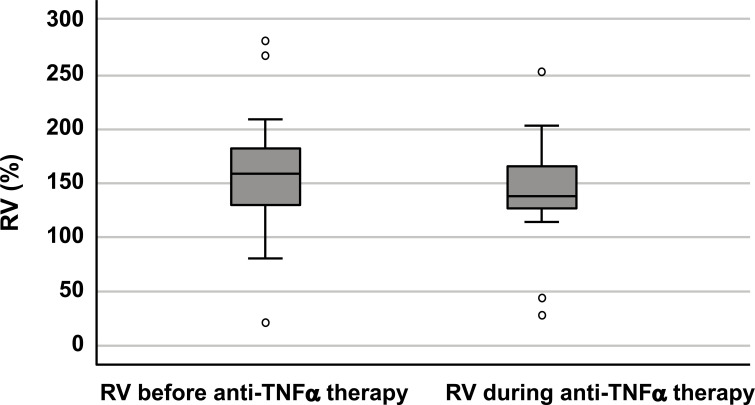
Comparison of residual volume relative before and during anti-TNFα therapy. Median residual volume relative before anti-TNFα therapy is 159% (IQR 129.25 - 184.75), residual volume relative during anti-TNFα therapy is 143% (IQR 127.00 - 165.25), *p*=0.016.

**Fig. (2):**
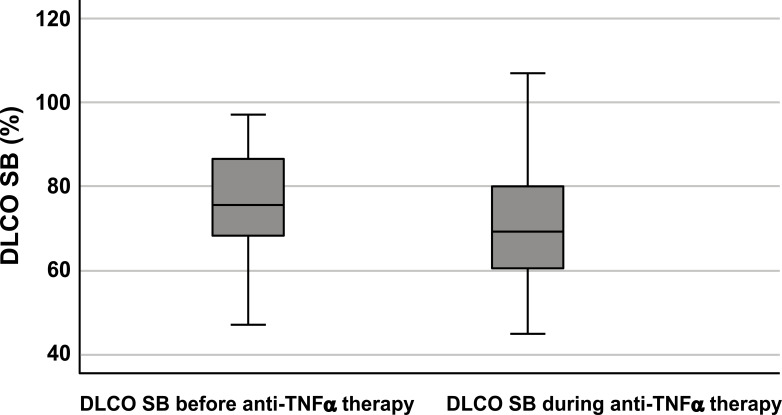
Comparison of DLCO before and during anti-TNFα therapy. A non-significant reduction in DLCO SB during anti-TNFα therapy is to be noted (from a mean value of 76.1% to 72.0% during anti-TNFα therapy).

In the anti-TNFα therapy follow-up group, only residual volume improved during TNFα therapy, with significant increases in both absolute and relative values (Fig. **1**). A non-significant reduction in DLCO SB during anti-TNFα therapy must be noted (from a mean value of 76.1% to 72.0% during anti-TNFα therapy) (Fig. **2**). The remaining parameters presented no significant changes. The residual volume was observed to be above the physiological range both before and during anti-TNFα therapy. The DLCO was observed to be at the lower end of the normal range prior to the initiation of therapy and demonstrated a slight, non-significant reduction during the course of therapy. The moderately increased mean residual volume decreased during the course of therapy, indicating improvement (absolute residual volume: 2.7 L [IQR 2.04–3.0^6^] before anti-TNFα therapy *vs.* 2.5 L [IQR 2.14–3.00] during anti-TNFα therapy, *p* = 0.031; relative residual volume: 159% [IQR 129.25–184.75] before anti-TNFα therapy *vs.* 143% [IQR 127.00–165.^25^] during anti-TNFα therapy, *p* = 0.016) (Fig. **1**) Twenty two of the 32 patients showed a reduction. The other parameters remained within the physiological range before and during anti-TNFα therapy and therefore presented no discernible change. All lung function parameters are available in the **Supplementary Material**.

## 4 DISCUSSION

The aim of this clinical study was to evaluate the pulmonary involvement of patients with IBD and the influence of anti-TNFα therapy on lung function parameters. Therefore, the lung functions of patients with IBD were examined before and during anti-TNFα treatment.

This study failed to show a benefit of anti-TNFα therapy regarding the most relevant lung function test results. The mean values for FEV1 and VC, as determined by lung function tests prior to the commencement of anti-TNFα therapy and throughout the course of therapy, were found to be within the normal range. Therefore, there was no evidence of a significant obstructive or restrictive ventilation disorder.

The residual volume was moderately increased both before and during anti-TNFα therapy. Even though mean values for FEV1 and VC before and during anti-TNFα therapy were found to be within the normal range, the isolated pathological values of residual volume may indicate the presence of a clinically relevant respiratory disease component [28]. The residual volume indicates the remaining air in the lungs after maximum expiration. Increased residual volume means air trapping. Air trapping is related to obstructive ventilation disorders like asthma or COPD. In our study, the residual volume showed a small but significant improvement during anti-TNFα therapy. The isolated improvement in residual volume during anti-TNFα therapy may indicate a positive effect on a mild underlying obstructive ventilation disorder.

The diffusion factor (DLCO SB) was initially slightly below the lower normal range. This may be interpreted as a pulmonary extraintestinal manifestation of IBD. Contrary to our expectations, there was an even further decrease in the diffusion capacity during anti-TNFα therapy, although not significant.

Correlatively with our study, a DLCO SB reduction in IBD patients has already been described; therefore, this value was considered to indicate latent lung involvement in IBD in the presence of otherwise normal VC and FEV1 [29]. Diffusion disorder is known to be related to the degree of disease activity [14, 28]. An expected improve-ment in diffusion disorder during anti-inflammatory therapy with anti-TNFα was not observed in our study.

All values in this study should be interpreted in the context of prior anti-inflammatory medication. Our patient cohort was not medication native. The analyzed patient group had received different therapies for inflammatory bowel disease, including systemic steroid therapy, before the commencement of anti-TNFα therapy, which may have influenced the initial lung function tests.

The aim of pharmacological therapy for IBD is to initiate and maintain remission of the bowel disease. According to the literature, remission of IBD is associated with improved lung function compared to the activated disease state [30]. In patients with long-term untreated IBD, we would have expected worse pulmonary function parameters according to the increased disease activity [10]. Thus, almost unchanged lung function parameters in our study cohort compared to untreated patients can already be expected as a result of successful previous treatment of the bowel disease. This can lead to an underestimation of the pulmonary effect of anti-TNFα therapy. Otherwise, the significant improvement in residual volume and the non-significant worsening of diffusion capacity may be related to the cessation of previous IBD-related therapy instead of the effect of the anti-TNF therapy.

Nevertheless, we could demonstrate pulmonary function impairment in patients with inflammatory bowel disease and a slight isolated improvement in residual volume during anti-TNFα therapy, which, however, remains without clear clinical relevance.

## CONCLUSION

Our study patients had only minor impairment in lung function before anti-TNFα therapy. Mean residual volume (RV) was moderately increased, and diffusion capacity was slightly reduced. During anti-TNFα therapy, lung function parameters slightly changed, at least in patients with mild IBD.

## Figures and Tables

**Table 1:** Epidemiological data of the patients included in the study.

**Patient Group**	**Amount/age**
Number of all patients	32
Mean age of all patients	33y
Male	20
Female	12
Colitis ulcerosa	14
Crohn's disease	18

## Data Availability

The data and supportive information are available within the article.

## LIST OF ABBREVIATIONS

VC: Vital Capacity
RV: Residual Volume
IBD: Inflammatory Bowel Disease
FEV: Forced Expiratory Volume
MCID: Minimal Clinically Important Difference
